# Towards Malaria Elimination: A Nationwide Case–Control Study to Assess Risk Factors for Severe Malaria‐Related Deaths in Brazil

**DOI:** 10.1111/tmi.70028

**Published:** 2025-09-24

**Authors:** Klauss K. S. Garcia, Gabriel Z. Laporta, Elisabeth Carmen Duarte, Seyi Soremekun, Amanda Amaral Abrahão, Anderson Coutinho da Silva, Anielle de Pina Costa, Marcus Vinícius Guimarães Lacerda, Chris Drakeley, Walter Massa Ramalho, André M. Siqueira

**Affiliations:** ^1^ Centre for Tropical Medicine University of Brasilia Brasilia Brazil; ^2^ Department of Infectious Disease Epidemiology and International Health, Faculty of Epidemiology and Population Health The London School of Hygiene & Tropical Medicine London UK; ^3^ Graduate Program in Health Sciences FMABC Medical School University Centre Santo André São Paulo Brazil; ^4^ Department of Infection Biology The London School of Hygiene & Tropical Medicine London UK; ^5^ Faculty of Health Sciences University of Brasilia Brasilia Brazil; ^6^ Fluminense Federal University Niterói Rio de Janeiro Brazil; ^7^ Fundação de Medicina Tropical Doutor Heitor Vieira Dourado Manaus Brazil; ^8^ Evandro Chagas National Institute of Infectious Diseases Fundação Oswaldo Cruz (Fiocruz) Rio de Janeiro Rio de Janeiro Brazil

**Keywords:** deaths, epidemiological surveillance, malaria, public health, record linkage, risk factors

## Abstract

**Objective:**

The objective of this study is to describe malaria‐related deaths and assess their risk factors.

**Methods:**

This is a case–control study using data from Brazil's Ministry of Health (2011–2020) on malaria‐related deaths (ICD‐10: B50‐B54). A probabilistic record linkage was performed to match epidemiological data (Sivep‐Malaria and Sinan) with death records from the Mortality Information System (SIM). Cases were defined as individuals who died of malaria, and controls were malaria cases that did not result in death. Logistic regression models were used to identify the factors associated with malaria mortality.

**Results:**

A total of 632 malaria‐related deaths were recorded, with 454 (71.8%) occurring in the Amazon region and 178 (28.1%) outside it. Risk factors in the Amazon included age (< 6 months or > 60 years; *p* ≤ 0.01), delayed treatment (> 48 h; *p* < 0.001), illiteracy (*p* = 0.01), and living in indigenous population villages (*p* < 0.001), while active case detection was protective (*p* = 0.01). In the Extra‐Amazon, risk factors included delayed treatment (*p* = 0.049), *P. falciparum* or mixed infections (*p* < 0.049), foreign‐acquired infections (*p* = 0.01), and higher education level (*p* = 0.03)—which is a proxy for increased income and travel frequency, which may increase the likelihood of exposure in endemic areas and delayed diagnosis upon return.

**Conclusion:**

This nationwide record‐linkage study shows that malaria deaths remain concentrated in socially vulnerable groups: infants, older adults, and especially indigenous populations living in the Amazon. Delayed treatment is also a determinant for deaths, in both endemic and non‐endemic regions, while active case detection markedly reduces the odds of death. To reach Brazil's zero‐malaria‐death target by 2030, it is needed to improve timely diagnosis and treatment, to enhance epidemiological information systems along with active surveillance amplification in remote communities. Finally, integrating national health‐information systems allows real‐time monitoring, and with coordinated action, eliminating malaria deaths remains achievable.

## 1 Introduction

Malaria is an acute infectious disease characterised by fever which primarily affects tropical and developing countries and, if not promptly diagnosed and treated, can rapidly progress to death. According to the World Health Organization (WHO), over 263 million people were affected by malaria, and over 597,000 died in 2023 [1]. Within the broader framework of the Sustainable Development Goals (SDGs), the international community set ambitious targets to combat malaria. In 2015, the WHO published the Global Technical Strategy for Malaria 2016–2030 (GTS), which provides a comprehensive framework aimed at reducing malaria incidence and mortality rates by at least 90% by 2030. The strategy emphasises universal access to prevention, diagnosis, and treatment, along with strengthening health systems to sustain malaria control progress [2].

Despite ongoing global efforts to control and eliminate malaria, progress has slowed in high‐burden countries. Globally, malaria mortality rates range from 0.2% to 2.2% [3]. To accelerate progress towards malaria elimination, Brazil's Ministry of Health (BMoH) National Malaria Program launched the National Malaria Elimination Plan in 2022, which outlines a plan to eliminate malaria‐related deaths by 2030 and autochthonous cases by 2035 [4].

In Brazil, the primary *Plasmodium* species responsible for malaria transmission are *P. vivax* , responsible for approximately 83.7% of cases in the region, and *P. falciparum*, responsible for 16.3% [5]. Interrupting malaria transmission from confirmed cases is critical, and this is achieved through the administration of effective antimalarial drugs to eliminate the parasite from the body. Malaria diagnosis and treatment in Brazil are performed by the Unified Health System (SUS), free of charge to the patient. Proper diagnosis is essential to provide opportune treatment and prevent deaths [6].

Brazil currently is the country with the most malaria cases in the WHO Americas Region [1], and its Amazon region accounts for approximately 99% of the country's malaria cases. In contrast, the Extra‐Amazon (not endemic) region presents a small portion of cases with higher lethality [7]. The higher mortality observed in the Extra‐Amazon region is mainly due to delayed diagnosis and low clinical suspicion, since malaria is uncommon in that area. Health professionals and patients often fail to consider malaria as an initial diagnosis, leading to confusion with other endemic febrile illnesses such as dengue. This delay in diagnosis and treatment—especially among individuals infected elsewhere and diagnosed only upon return—significantly increases the risk of progression to severe and fatal outcomes [8].

In 2023, Brazil registered over 142,000 new malaria infections and 80 malaria‐related deaths [9, 10]. Although the number of deaths is low relative to the total number of cases, these fatalities highlight structural weaknesses within Brazil's SUS, as malaria‐related deaths are considered preventable [11]. International studies have identified several factors such as demographic factors, pre‐existing medical conditions, delay in diagnosis and treatment, travel history to endemic regions, and *P. falciparum* infection that contribute to malaria‐related deaths [12, 13, 14, 15, 16, 17].

Despite malaria remaining a major public health issue in—and with national targets calling for the elimination of malaria cases and deaths by 2035 [18, 19]—robust evidence on the determinants of malaria mortality is still scarce. This knowledge gap is a result of the lack of integration between Brazilian malaria surveillance databases and the Brazilian death records system; their data remain fragmented, and current malaria information systems do not track patient outcomes, so currently it is not feasible to know whether a patient with malaria progressed exclusively to cure or to a relapse, hospitalisation, or death [20]. No previous study has analysed death‐associated factors nationwide using longitudinal, comparative methods, nor applied probabilistic record linkage to overcome the lack of interoperability among health information systems (HIS).

Therefore, this study objective is to identify the factors associated with malaria‐related deaths across Brazilian territory and provide specific recommendations to support Brazil's goal of zero malaria deaths by 2030.

## 2 METHODS

### 2.1 Study Design, Period, Population, and Location

**FIGURE 1:**
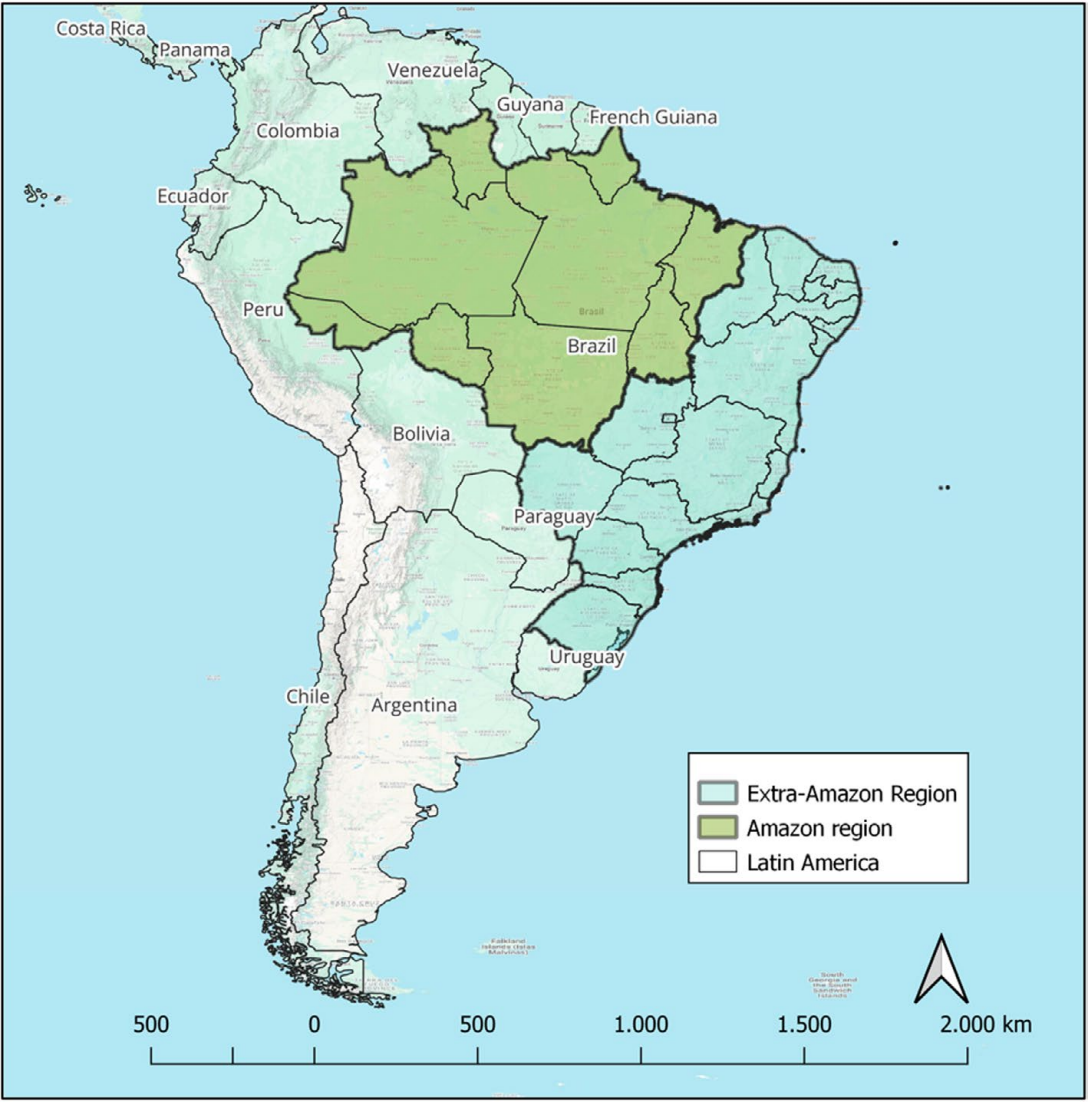
Brazil's location and its regions. *Source*: Google Maps.

Considering that malaria deaths are an infrequent outcome from malaria infections, a case–control study was designed utilising surveillance data from the BMoH from 2011 to 2020. It analysed malaria‐related deaths (ICD‐10: B50–B54) and malaria infection cases reported across the entire Brazilian territory, encompassing both the Amazon region and the Extra‐Amazon region (Figure 1).

The Amazon region is characterised by high incidence rates of malaria. Its extensive tropical forests, rivers, rain cycle and other natural environments favour the proliferation of *Anopheles* mosquitoes, the primary vectors of malaria. Additionally, the socio‐economic profile of the local communities is marked by limited access to healthcare services, poverty, low educational levels, and dependence on agriculture, fishing, and extractive activities, which often bring individuals into close contact with forested areas where malaria transmission is highest [8]. Furthermore, indigenous populations, who constitute a significant portion of the Amazon region's demographic, are particularly vulnerable due to cultural practices and subsistence activities [21, 22].

In contrast, the Brazilian Extra‐Amazon region experiences much lower malaria incidence rates, mostly due to imported cases. Malaria cases in this region are typically caused by *P. vivax* infections, but with a higher proportion of *P. falciparum* cases compared to the Amazon region. Additionally, health surveillance systems in the Extra‐Amazon region are less vigilant in detecting malaria cases. The population in this region is generally more urbanised, with higher levels of education and better access to healthcare compared to the Amazonian population [8].

Additionally, this study did not involve sample size or power calculations, as it was based on a census of all available data. We included all malaria‐related deaths and all non‐fatal malaria cases reported in national surveillance systems between 2011 and 2020.

### 2.2 Data Source and Record Linkage Procedures

Data access was authorised by the BMoH (protocol #25000.052402/2024‐04, Supporting Information I). Three national HIS were used: the Malaria Epidemiological Surveillance System (Sivep‐Malaria) for malaria cases recorded in the Amazon region, the Notifiable Diseases Information System (Sinan) for malaria cases recorded in the Extra‐Amazon region, and the Mortality Information System (SIM) for malaria‐related deaths.

A probabilistic record linkage (RL) was performed to integrate these datasets, following data cleaning protocols previously established by our group [23, 24]. RL was conducted in R programming language (version 4.2.1) using the ‘fuzzyjoin’ package [25]. The matching algorithm applied Levenshtein and Cosine distance metrics, using thresholds of 3 and 0.5, respectively [20].

These parameters were selected to balance sensitivity and specificity, considering the frequent typographical inconsistencies in Brazilian HIS. A Levenshtein threshold of three‐character mismatches increases the likelihood of capturing true matches affected by data entry errors, while the Cosine similarity threshold of 0.5 is a commonly accepted standard for textual matching. To further ensure the accuracy of the linkage process, all matched pairs underwent manual review to exclude false positives and validate true matches.

Key linkage variables included patient's name, date of birth, and mother's name [23, 26], and to improve match quality, all names were pre‐processed by removing connectors (e.g., ‘da’, ‘de’, ‘do’), accents, punctuation, numbers, and extraneous text (e.g., ‘PESO’, ‘TEL’, ‘unknown’). The ‘Patient's Name’ field was split into first and last names to enhance sensitivity.

Additionally, only records where the date of malaria symptom onset preceded the date of death were retained. Pairs were excluded if death occurred more than 365 days after symptom onset or more than 30 days before it, to account for possible notification errors.

### 2.3 Cases and Controls Definition

Cases were individuals registered in the Mortality Information System for malaria‐related deaths that were true matches after the RL process. Controls are all malaria infections that did not have corresponding pairs in the RL process; therefore, malaria infections that did not result in death.

We defined and *analysed* three sets of case–control groups, namely: (I) Group I (Amazon Region)—cases and controls were reported in the Amazon region; (II) Group II (Extra‐Amazon region)—cases and controls were reported in the Extra‐Amazon region; and (III) Group III (Brazil)—cases and controls were reported in the Brazil, regardless of whether they were reported in the Amazon or Extra‐Amazon region.

### 2.4 Variables

Study variables are from different surveillance databases, namely: Mortality Information System (SIM), Epidemiological Surveillance Information System for Malaria (Sivep‐Malaria), and Notifiable Diseases Information System (Sinan).

Variables *analysed* are: sex, age range, skin colour, education level (in years of schooling), place of death, underlying and associated causes of death (death caused by *P. vivax* infections or *P. falciparum*, or other malaria parasite species, and malaria parasite species not specified—NS), notification and symptoms onset date, treatment date, occupation in the last 15 days, presumed site of infection, country of infection, means of detection (active detection—which is when health professionals visit communities conducting housing surveys to detect people presenting symptoms, or conducting testing and treatment strategies; or passive detection—when the patients seek care at health units by themselves).

To perform the logistic regression analysis, some variables were aggregated or categorised, as follows.

The variable ‘skin colour’ was categorised into three groups: black (database fields: ‘black’ and ‘brown’ options), indigenous, and white (database fields: ‘white’ and ‘yellow’).

The variable ‘occupations in the last 15 days’—which specifies what type of occupation the patient conducted in the past 15 days—was categorised into five different categories: ‘agriculture’ (database fields: ‘agriculture’, ‘hunting/fishing’, ‘plant exploitation’, and ‘livestock’), ‘travel related’ (database fields: ‘tourism’ and ‘traveller’), ‘mining’ (database fields: ‘Garimpagem’, and ‘Mining’), ‘housing’, and ‘others’ (database fields: ‘others’, ‘driver’, and ‘road/dam construction’). For the Extra‐Amazon region analysis, it was used only ‘other’ occupations and ‘travel related’ occupations were used, due to a reduced number of observations.

The variable ‘treatment opportunity’ was calculated using the interval—in days—between the date of treatment and the symptoms' onset date for each patient. It was then categorised into two groups: treatment within 48 h and treatment after 48 h.

The exam results (diagnosis) were classified into: *P. falciparum and* mixed infection (database fields: 2—*Pf*; 3—*Pf* + fg; 5—*Pf* + *Pv*; 6—*Pv* + fg; 7—fg; 9—*Pf* + *Pm*); *P. vivax* (database fields: 4— *P. vivax* ; 11—non‐falciparum); and other species (database fields: 8—*P. malariae*; 10— *P. ovale* ).

Deaths from imported malaria (infected outside Brazil) were classified according to the information in the ‘Country of infection’ variable. All countries different from ‘Brazil’ (code: 001) were classified as ‘imported’, and those infected in ‘Brazil’ as ‘autochthonous’.

### 2.5 Statistical Analysis

Descriptive analyses were carried out to characterise the research subjects. Accumulated lethality rates were calculated by dividing the number of malaria‐related deaths by the number of malaria cases (×100).

A spatial distribution of malaria‐related deaths using *heat maps* was generated using kernel density estimation with weighted data points. Weights were based on the number of deaths per municipality, using geographic centroids to define spatial proximity. The analysis was conducted in QGIS software, applying default bandwidth and smoothing parameters.

Additionally, we assessed diagnostic consistency between the mortality system (SIM) and the epidemiological surveillance systems (Sivep‐Malaria and Sinan) using Cohen's kappa. Both the unweighted kappa (treating all disagreements equally) and a custom weighted kappa were calculated. For the weighted analysis, we specified a 4 × 4 weight matrix (categories: *Pv*, *Pf*, Mixed, Other; all ‘Unspecified’ entries zero) in which:

- Perfect matches (*Pv*–*Pv*, *Pf*–*Pf*, Other–Other) were assigned weight = 1.
- Severe misclassifications (*Pv*–*Pf, Pf*–*Pv*, any vs. Unspecified) were assigned weight = 0.
- Moderate misclassifications involving mixed infections or ‘other’ forms (*Pv*–Mixed, *Pf*–Mixed, Other–Mixed) were assigned weight = 0.5.
- All other mismatches (*Pv*–Other, *Pf*–Other, Other–*Pv*, Other–*Pf*) were assigned a weight of 0.

This scheme reflects the clinical importance of distinct species discrepancies and yields a more nuanced measure of agreement than the unweighted statistics. Continuous Kappa estimates and 95% confidence intervals, as well as a formal *p* value test of *κ* = 0 versus *κ* ≠ 0, are reported for each region [26].

In the case–control analysis, chi‐square tests were used to assess the associations between exposed variables and outcome (malaria‐related death), prior to their inclusion in the logistic regression model. Only variables with a *p* value under 0.20 in the chi‐square test were incorporated into the logistic regression models [27]. We assessed multicollinearity among all candidate predictors and found no variable exhibiting problematic correlation, so all were retained in the models. Missing data were described in the descriptive analysis and no record containing NA values in its variables was excluded.

Logistic regression models were employed to identify associated factors for malaria‐related deaths. Initially, univariate logistic regressions were conducted to calculate the crude odds ratio, followed by the inclusion of a full model, and then advancing to the adjusted model using a backward selection method. A significance level of 5% was applied to all models.

### 2.6 Software

The following software was utilised for data processing and analysis: R version 4.2.1, Microsoft Excel 2016, QGIS version 3.28, and Jamovi version 2.4.11.0.

### 2.7 Ethical Considerations

This project received approval from the Research Ethics Committee of the Faculty of Medicine at the University of Brasília in January 2022, under protocol CEP: 5,216,688 (51246121.0.0000.5558). The use of secondary, identifiable data from national HIS was authorised without the need for informed consent, as permitted under Resolution No. 510/2016 of the Brazilian National Health Council (Article 14). Identifiable data were accessed exclusively within a secure environment of the BMoH for the purpose of conducting record linkage. After the linkage process, all datasets were de‐identified and anonymised before analysis, following strict confidentiality and data protection protocols.

## 3 Results

### 3.1 Time and Spatial Behaviour

**FIGURE 2:**
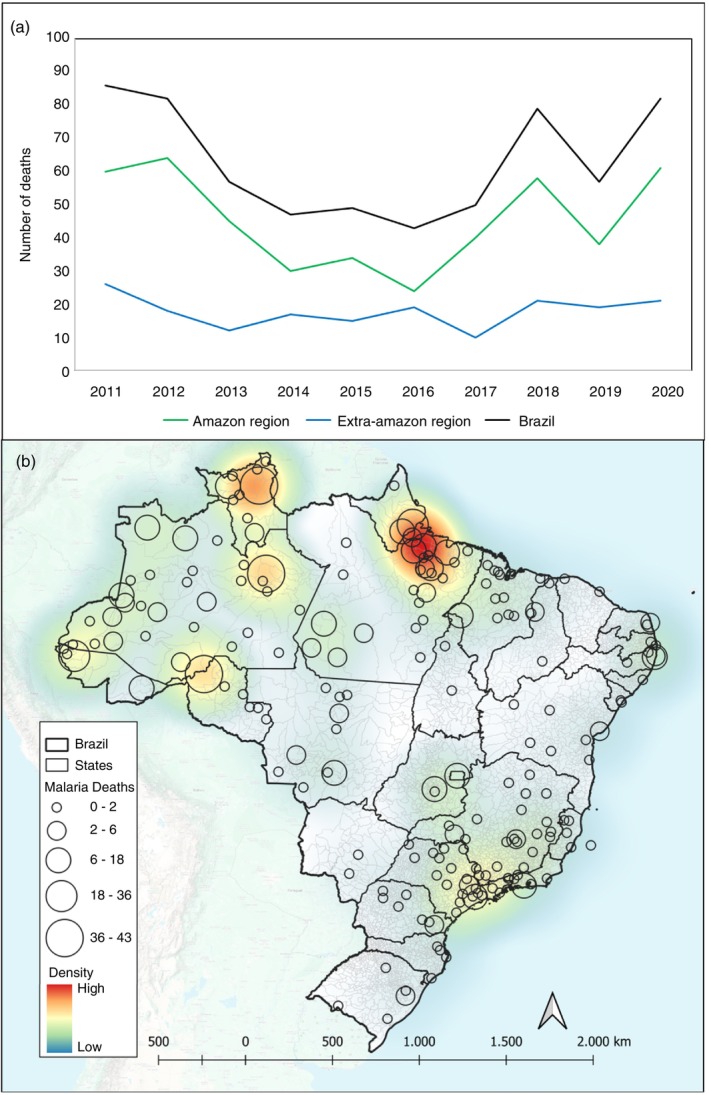
Malaria‐related deaths in Brazil, 2011–2020. (a) Total number of malaria‐related deaths by region of occurrence in Brazil (Amazon and Extra‐Amazon regions), from 2011 to 2020. (b) Spatial distribution and density of malaria‐related deaths across Brazil from 2011 to 2020.

During the study period, 632 malaria deaths were recorded, with 454 (71.8%) occurring in the Brazilian Amazon region and 178 (28.1%) in the Extra‐Amazon region. A decrease in the number of deaths was observed in Brazil, particularly in the Amazon region, between 2011 and 2016. Since then, there has been an increase, reaching 82 deaths in 2020. The cumulative case‐fatality rate in the Amazon region was 0.02%, while in the Extra‐Amazon region it was 2.52%, making the latter 115.6 times the case‐fatality rate in the Amazon region (Figure 2a).

The spatial distribution of deaths shows higher density in the northern region of the country. Notably, deaths were recorded in the country's border regions, forming a ‘belt of mortality’ along the borders with neighbouring countries. In the Extra‐Amazon region, deaths occurred more frequently in the capitals of each state, particularly in the Southeast, where São Paulo and Rio de Janeiro are located (Figure 2b).

### 3.2 Epidemiological Profile

The proportional distribution of deaths shows a predominance of men (*N* = 371, 58.7%), over 60 years of age (*N* = 210, 33.2%), black (*N* = 374, 59.2%), and with low or no education (7 years of schooling or less) (*N* = 309, 49.0%). Most deaths occurred in a hospital setting (*N* = 490, 77.5%), but only 126 (19.9%) were formally investigated. The most frequent diagnosis was *P. vivax* (*N* = 255, 40.3%), followed by ‘malaria NS’ (*N* = 226, 35.8%) and *P. falciparum* (*N* = 129, 20.4%).

Key differences between deaths profile reported in the Extra‐Amazon region—compared to the Amazon region—were a lower proportion of young people (under 20 years of age) and higher proportions of white individuals, with higher education (over 11 years) and unidentified *Plasmodium* species (Table 1).

**TABLE 1:** Malaria‐related deaths epidemiological profile in Brazil, 2011–2020.

	Amazon region	%	Extra‐Amazon region	%	Total	%
Total	454	71.8	178	28.2	632	100.0
Sex						
Male	269	59.3	102	57.3	371	58.7
Female	185	40.7	76	42.7	261	41.3
Age range						
Under 6 months old	19	4.2	1	0.6	20	3.2
6–11 months old	10	2.2	0	0.0	10	1.6
1–4 years old	33	7.3	1	0.6	34	5.4
5–9 years old	19	4.2	0	0.0	19	3.0
10–19 years old	36	7.9	5	2.8	41	6.5
20–39 years old	98	21.6	43	24.2	141	22.3
40–59 years old	101	22.2	55	30.9	156	24.7
60 years old and over	137	30.2	73	41.0	210	33.2
Unknown	1	0.2	0	0.0	1	0.2
Race/Colour of skin						
White	71	15.6	89	50.0	160	25.3
Black	295	64.9	79	44.4	374	59.2
Indigenous	83	18.3	0	0.0	83	—
Unknown	5	1.1	10	5.6	15	2.4
Education level (years of schooling)						
None	95	20.9	16	9.0	111	17.6
1–7 years	155	34.2	43	24.2	198	31.4
8–11 years	62	13.7	44	24.7	106	16.8
12 years and over	21	4.6	41	23.0	62	9.8
Unknown	121	26.6	34	19.1	155	24.4
Local of death						
Hospital	343	75.6	147	82.6	490	77.5
Other health facilities	8	1.8	6	3.4	14	2.2
At home	68	15.0	22	12.4	90	14.2
On the street	11	2.4	1	0.6	12	1.9
Others	24	5.3	2	1.1	26	4.1
Was the death investigated?						
Yes	78	17.2	48	27.0	126	19.9
No	221	48.7	76	42.7	297	47.0
Unknown	155	34.1	54	30.3	209	33.1
Was malaria the main cause of death?						
Yes	336	74.0	117	65.7	453	71.7
No	118	26.0	61	34.3	179	28.3
Diagnosis						
*Plasmodium vivax*	235	51.8	20	11.2	255	40.3
*Plasmodium falciparum*	82	18.1	47	26.4	129	20.4
Other species	6	1.3	16	9.0	22	3.5
Unspecified malaria	131	28.9	95	53.4	226	35.8

Among the 632 recorded deaths, 71.6% had malaria diagnosed as the primary cause of death, with 12 deaths involving HIV co‐infection (ICD‐10 codes B20–B24). Of the 82 malaria‐related deaths that occurred in 2020, 15 deaths involved co‐infection with COVID‐19 (ICD‐10 codes B34 and U07). The most frequently reported symptoms were ‘Other sepsis’ (ICD‐10 A41) (*N* = 147; 23.3%), ‘Respiratory failure’ (ICD‐10 J96) (*N* = 110; 17.4%), and ‘Other symptoms and signs involving the circulatory and respiratory systems’ (ICD‐10 R09) (*N* = 82; 13.0%).

### 3.3 Linkage Results

In the Amazon region, the record linkage process involved 454 malaria‐related deaths and 2,128,409 reported malaria cases. After removing duplicate notifications and linkage duplicates (*N* = 48,134), a total of 188 deaths (29.7%) were successfully linked (case group I), along with 2,080,087 non‐fatal malaria cases (control group I).

In the Extra‐Amazon region, 178 deaths and 7147 reported malaria cases were analysed. After excluding duplicates (*N* = 6), 89 deaths (14.1%) were matched (case group II), alongside 7052 non‐fatal cases (control group II).

**FIGURE 3:**
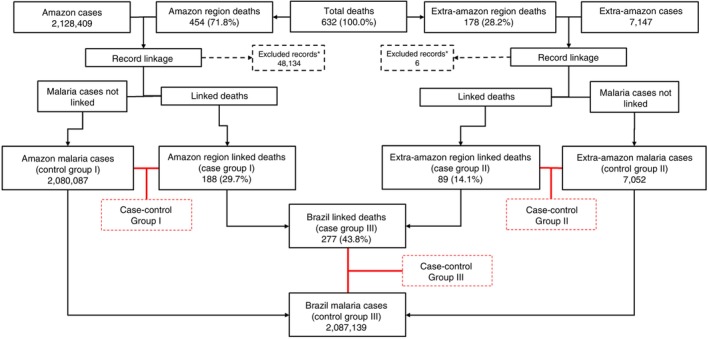
Flowchart with the description of the Record Linkage process and selection of case–control groups. *Exclusions due to: duplicated records; deaths that happened 1 year or over after the first infection; deaths reported in the health information systems over 30 days after it happened.

Overall, 277 deaths (43.8%) were successfully linked across both regions (case group III), along with 2,087,139 non‐fatal malaria cases (control group III), as shown in Figure 3.

### 3.4 Consistency Analysis

Among the 277 linked deaths, 92 (33.2%) were attributed to ‘unspecified malaria’. After comparing surveillance data using mortality and epidemiologic information systems, it was possible to identify that 54 deaths (58.7%) were due to *P. vivax* infections and 30 deaths (32.6%) were caused by *P. falciparum* infections. In the Amazon region, 88% of the ‘unspecified malaria’ deaths were due to *P. vivax* infections, whereas in the Extra‐Amazon region, 71.4% were due to *P. falciparum* or mixed infections. The consistency between species identification in mortality and epidemiological records was very high for *P. vivax* cases (94.9%), but lower for *P. falciparum* cases (69.5%) (Table 2).

**TABLE 2:** Consistency analysis between epidemiological surveillance systems diagnosis and cause of death diagnosis in the mortality system.

Region	Species in death certificate	Species in epidemiological system									
		Pv	%	Pf	%	Mixed	%	Other forms	%	Total deaths	%
Brazil	*Pv*	111	94.9	4	3.4	2	1.7	0	0.0	117	42.2
	*Pf*	12	20.3	41	69.5	5	8.5	1	1.7	59	21.3
	Other forms	3	33.3	3	33.3	2	22.2	1	11.1	9	3.2
	Unspecified malaria	54	58.7	30	32.6	6	6.5	2	2.2	92	33.2
Total (Brazil)		180	65.0	78	28.2	15	5.4	4	1.4	277	100.0
Amazon region	*Pv*	102	95.3	4	3.7	1	0.9	0	0.0	107	56
	*Pf*	12	41.4	15	51.7	2	6.9	0	0.0	29	15.2
	Other forms	3	60.0	1	20.0	1	20.0	0	0.0	5	2.6
	Unspecified malaria	44	88.0	6	12.0			0	0.0	50	26.2
Total (Amazon region)		161	84.3	26	13.6	4	2.1	0	0.0	191	100.0
Extra‐Amazon region	*Pv*	9	90.0			1	10.0	0	0.0	10	11.6
	*Pf*			26	86.7	3	10.0	1	3.3	30	34.9
	Other forms			2	50.0	1	25.0	1	25.0	4	4.7
	Unspecified malaria	10	23.8	24	57.1	6	14.3	2	4.8	42	48.8
Total (extra‐AMZ region)		19	22.1	52	60.5	11	12.8	4	4.7	86	100.0

Abbreviations: *Pf*, *Plasmodium falciparum*; *Pm*, *Plasmodium malariae*; *Pv*, *Plasmodium vivax*.

**FIGURE 4:**
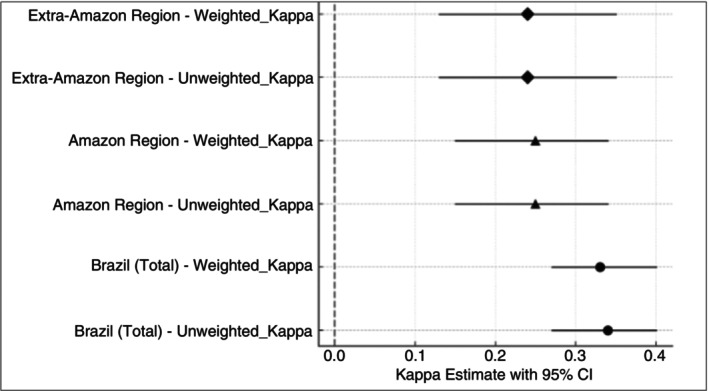
Kappa estimates for species‐diagnosis consistency between the Mortality System and Epidemiological Surveillance Systems.

The Cohen's *κ* coefficients for the country level consistency analysis presented an unweighted *κ* of 0.34 (95% CI: 0.27–0.40) and the weighted *κ* was 0.33 (95% CI: 0.27–0.40), both highly significant (*p* < 0.001). For the Amazon region, the unweighted and weighted *κ* values were each 0.25 (95% CI: 0.15–0.34; *p* < 0.001), and in the Extra‐Amazon region both were 0.24 (95% CI: 0.13–0.35; *p* < 0.001). These *κ* statistics confirm a minimal level of diagnostic consistency across systems, highly influenced the high amount of ‘Unspecified malaria’ diagnostics in the Mortality system (Figure 4).

### 3.5 Linked Deaths Profile

Among the 277 linked deaths, the majority were male (*N* = 171, 61.7%), aged between 20 and 39 years (*N* = 87, 31.4%), black individuals (*N* = 157, 56.7%), and had low educational attainment (under 7 years of schooling) (*N* = 106, 38.3%). The most frequent occupations were categorised as ‘other’ (unspecified) (*N* = 116, 41.9%), followed by rural activities (*N* = 49, 17.7%). A high proportion of individuals were infected in rural areas (*N* = 84, 30.3%) and indigenous population villages (*N* = 39, 14.1%).

A relevant proportion of malaria deaths (24.9%) was of people who possibly became infected in other countries (imported cases), particularly among the deaths reported in the Extra‐Amazon region (66.3%) (Table 3).

**TABLE 3:** Epidemiological profile of malaria‐related deaths according to region, Brazil, 2011–2020.

	Amazon region, *N* = 188	%	Extra‐Amazon region, *N* = 89	%	Brazil, *N* = 277	%
Sex						
Male	110	58.5	61	68.5	171	61.7
Female	78	41.5	28	31.5	106	38.3
Age range						
Under 6 months old	8	4.3		0.0	8	2.9
6–11 months	4	2.1		0.0	4	1.4
1–4 years old	15	8.0	1	1.1	16	5.8
5–9 years old	8	4.3		0.0	8	2.9
10–19 years old	16	8.5	1	1.1	17	6.1
20–39 years old	51	27.1	36	40.4	87	31.4
40–59 years old	38	20.2	34	38.2	72	26.0
60 years old and over	48	25.5	17	19.1	65	23.5
Unknown	0	0.0		0.0	0	0.0
Race/Colour of skin						
White	19	10.1	36	40.4	55	19.9
Black	115	61.2	42	47.2	157	56.7
Indigenous	36	19.1	0	0.0	36	13.0
Unknown	18	9.6	11	12.4	29	10.5
Education level (years of schooling)						
None	30	16.0	1	1.1	31	11.2
1–7 years	68	36.2	7	7.9	75	27.1
8–11 years	45	23.9	14	15.7	59	21.3
12 years and over	7	3.7	25	28.1	32	11.6
Not applicable	25	13.3	1	1.1	26	9.4
Unknown	13	6.9	41	46.1	54	19.5
Occupation in the last 15 days						
Rural/Agriculture	40	21.3	9	10.1	49	17.7
Housing	24	12.8	3	3.4	27	9.7
Mining	17	9.0	5	5.6	22	7.9
Other	89	47.3	27	30.3	116	41.9
Travel related	5	2.7	37	41.6	42	15.2
Unknown	13	6.9	8	9.0	21	7.6
Local of infection						
Indigenous villages	39	20.7	0	0.0	39	14.1
Rural area	84	44.7	0	0.0	84	30.3
Urban area	29	15.4	0	0.0	29	10.5
Gold mining area	12	6.4	0	0.0	12	4.3
Unknown	24	12.8	89	100.0	113	40.8
Region of infection						
Amazon region	178	94.7	17	19.1	195	70.4
Extra‐Amazon region	0	0.0	5	5.6	5	1.8
Imported from another country	10	5.3	59	66.3	69	24.9
Unknown	0	0.0	8	9.0	8	2.9
Type of detection						
Passive detection	164	87.2	68	76.4	232	83.8
Active detection	22	11.7	17	19.1	39	14.1
Unknown	2	1.1	4	4.5	6	2.2
Treatment opportunity (hours since symptoms onset)						
≤ 48 h	44	23.4	9	10.1	53	19.1
> 48 h	91	48.4	72	80.9	163	58.8
Unknown	53	28.2	8	9.0	61	22.0
Diagnosis						
*P. vivax*	159	84.6	21	23.6	180	65.0
*P. falciparum* or mixed infection	29	15.4	64	71.9	93	33.6
Other forms	0	0.0	4	4.5	4	1.4

Most of the deaths were detected through passive surveillance (83.8%), and the majority with an important delay in treatment initiation (over 48 h after the onset of symptoms): 58.8% of all deaths, and 80.9% of those from the Extra‐Amazon region.

The leading cause of infection among the malaria‐related deaths was *P. vivax* in data from Brazil (65.0%) and from the Amazon region (84.6%) (Table 3). On the other hand, most deaths reported in the Extra‐Amazon region were caused by *P. falciparum* or mixed infections (71.9%).

### 3.6 Associated Factors Analysis by Region

**FIGURE 5:**
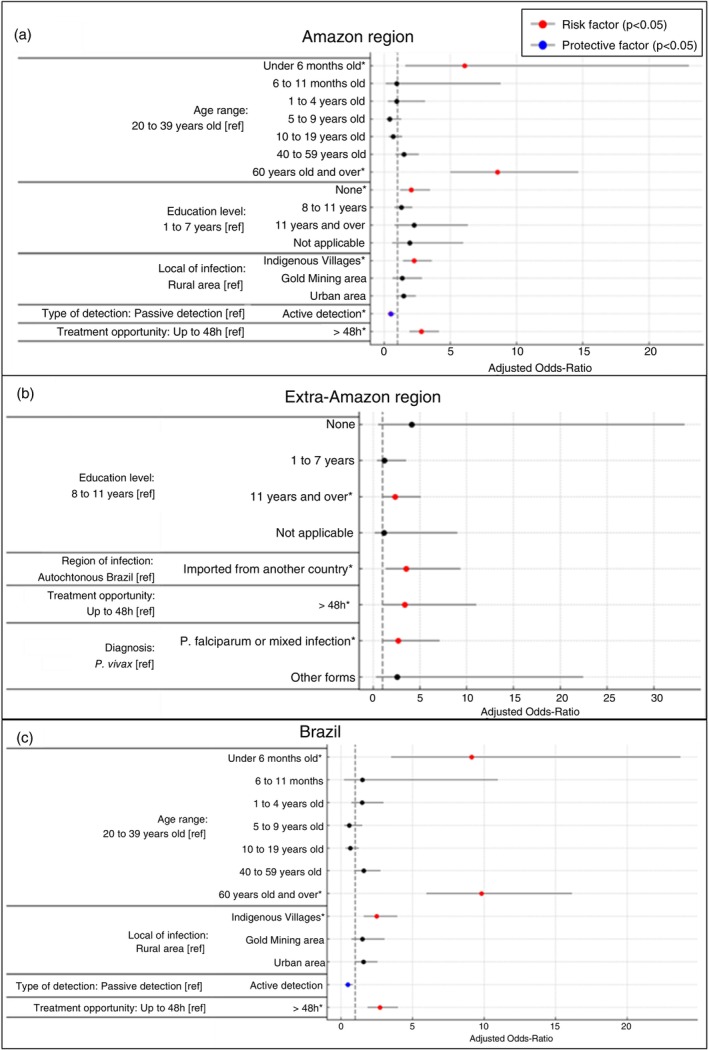
Adjusted odds ratio and confidence intervals for malaria deaths associated factors by region in Brazil, 2011–2020. (a) Brazilian Amazon region; (b) Brazilian Extra‐Amazon region; (c) Entire Brazil territory. Red dots mean statistically significant risk factors, and blue dots mean statistically significant protective factors.

In the Amazon region (case–control group I), the adjusted logistic regression model showed that active case detection was protective against malaria‐related death (AOR = 0.48; CI: 0.27–0.85; *p* = 0.01). Higher odds of death were found for infants < 6 months (AOR = 6.07; CI: 1.60–23.00; *p* = 0.01), adults ≥ 60 years (AOR = 8.56; CI: 5.01–14.65; *p* < 0.001), treatment initiation > 48 h after symptom onset (AOR = 2.81; CI: 1.90–4.15; *p* < 0.001), illiteracy (AOR = 2.05; CI: 1.21–3.46; *p* = 0.01), and infection acquired in indigenous villages (AOR = 2.26; CI: 1.42–3.60; *p* < 0.001) (Figure 5a).

In the Extra‐Amazon region (case–control group II), the adjusted model showed significantly higher odds of death for patients who began treatment more than 48 h after symptom onset (AOR = 3.35; CI: 1.02–11.03; *p* = 0.049), for infections caused by *P. falciparum* or mixed species (AOR = 2.67; CI: 1.01–7.10; *p* = 0.049), for individuals with > 11 years of education (AOR = 2.33; CI: 1.07–5.06; *p* = 0.03), and for cases acquired abroad (AOR = 3.52; CI: 1.33–9.32; *p* = 0.01) (Figure 5b).

In the nationwide model (case–control group III), active case detection remained protective (AOR = 0.47; CI: 0.27–0.83; *p* = 0.01). Higher odds of death were observed for infants < 6 months (AOR = 9.13; CI: 3.51–23.72; *p* < 0.001), adults ≥ 60 years (AOR = 9.81; CI: 5.96–16.14; *p* < 0.001), treatment initiation > 48 h after symptom onset (AOR = 2.72; CI: 1.86–3.99; *p* < 0.001), and infections acquired in indigenous villages (AOR = 2.49; CI: 1.58–3.93; *p* < 0.001) (Figure 5c).

The goodness‐of‐fit statistics were satisfactory for all three models. The Amazon‐region model, Extra‐Amazon model, and nationwide model had AIC values of 2341, 396, and 2404, respectively, and corresponding pseudo‐*R* ^2^ values of 0.06, 0.11, and 0.06. These metrics indicate an appropriate balance between model fit and parsimony; taken together, all three respective final models explain approximately 6%, 11%, and 6% of the odds variation in malaria mortality. Detailed tables with logistic regression results are available in Supporting Information II.

## 4 Discussion

### 4.1 General Scenario

This study is the first to apply probabilistic record linkage to identify associated factors for malaria‐related deaths in Brazil while offering a novel nationwide view of mortality determinants. The results highlight the main characteristics of malaria‐related deaths across Brazil, revealing that the spatial distribution of deaths is concentrated in major urban centres, including São Paulo/SP, Rio de Janeiro/RJ, Boa Vista/RO, Belém/PA, Manaus/AM, and Porto Velho/RR. Deaths occurred predominantly among males, older adults, Black individuals, and those with lower education.

There are significant regional differences that will be discussed in the following sections. In the Amazon region, *P. vivax* —the most prevalent species in Brazil [5]—accounts for most deaths, followed by unspecified diagnoses (ICD‐10: B54) and *P. falciparum* infections. The latter two are more commonly observed in the Extra‐Amazon region, reflecting diagnostic uncertainty, undertraining of clinicians in non‐endemic areas, underuse of confirmatory tests, and systemic underreporting of species‐specific data [8]. Deaths among children < 10 years are more frequent in the Amazon, consistent with the higher proportion of malaria cases in this age group (22.4% vs. < 3% in the Extra‐Amazon) [19, 25].

Areas with the highest density of malaria‐related deaths overlap with high‐risk classified municipalities [18, 19]. This underscores the need for intensified surveillance and preventive measures in high transmission risk areas. The higher lethality in the Extra‐Amazon region also suggests that while malaria is less prevalent in the Extra‐Amazon region, the health care services are inadequately prepared to swiftly detect and treat severe cases [8].

Malaria deaths are concentrated in the Amazon and disproportionately affect socially vulnerable populations who face barriers to timely, appropriate care [28]. Those populations often have limited knowledge about malaria prevention and may not recognise the early symptoms of the disease, leading to a delayed search for health care services. Additionally, geographic isolation, coupled with insufficient health infrastructure, exacerbates the risk of severe outcomes, as patients are unable to seek care promptly. Addressing the socio‐economic determinants of health and improving education on malaria prevention and early detection are critical steps towards reducing malaria mortality in these communities [29].

### 4.2 Record Linkage Results

The RL process matched 43.8% of all malaria‐related deaths included in this study, providing a comprehensive understanding of the mortality scenario. One of the main challenges of using RL techniques is to deal with poor data quality, particularly due to typographical errors in birth dates and misspellings of patients' and mothers' names across different systems, which likely limited the RL performance [20, 24].

The BMoH has been working towards unifying information systems under the ‘e‐SUS linha da vida’ project, an initiative that aims to consolidate various health information systems into one comprehensive system that will enable longitudinal tracking of individual health data throughout the life course [30].

The development of this system could facilitate the routine identification of patients with recurrent malaria infections, hospitalisations, or death. The absence of outcome fields in the malaria epidemiological surveillance systems, and the lack of interoperability between epidemiological surveillance and mortality (or hospitalisation) systems, underscores the reason why this study was conducted [20].

The results in Table 2 show that RL can improve malaria death investigations by clarifying causes of death, helping surveillance teams target actions, and preventing future deaths. The crude consistency between species diagnoses in epidemiological and mortality systems, especially for *P. vivax* infections, is a positive result of this study (Table 2). However, the level of misclassification of deaths due to *P. falciparum* infections as *P. vivax* is concerning.

Additionally, the *κ* coefficients indicated a minimal level of diagnostic consistency across mortality and epidemiological records—much influenced by the unspecified malaria diagnosis (ICD‐10: B54) [26], which poses serious challenges to effective malaria surveillance and case management. The low concordance undermines accurate tracking of species‐specific mortality and impedes targeted public health interventions. To address those gaps, it is imperative to strengthen the training of healthcare professionals, ensuring a reduction of misdiagnosis.

Moreover, this misclassification may have contributed to inadequate treatment, potentially influencing clinical outcomes. Further studies are warranted to investigate the impact of species misclassification on treatment efficacy and malaria mortality.

### 4.3 Associated Factors Analysis by Region

Based on the linked results and logistic regression analyses, it was possible to identify the risk factors for malaria‐related deaths that are predominant in the country, in its endemic, and in its non‐endemic regions. The risk factors identified in this study are consistent with historical reports on factors associated with severe malaria, such as delayed diagnosis and treatment [31], the type of *Plasmodium* (*P. falciparum*) [32, 33], as age‐related immune vulnerability [34], and infections originating from endemic locations [35].

### 4.4 The Amazon Region

Infants under 6 months of age and people over 60 years old were identified as a risk factor. This may be attributed to the low level of immunity in those ages [34]. Brazil continues to struggle with high infant mortality rates, particularly in socially vulnerable areas of the Amazon region [36].

The identification of indigenous village infections as a risk factor reflects the unique challenges of malaria management in these areas. Indigenous populations are particularly vulnerable due to geographic isolation, limited access to healthcare, cultural barriers, malnutrition, and inadequate basic sanitation, among other factors [37].

The proximity of indigenous areas to the Amazon rainforest, combined with activities such as deforestation and illegal gold mining [38, 39], creates an environment conducive to the proliferation of malaria vector mosquitoes [3]. Late diagnosis and treatment further increase the risk of death from malaria in indigenous populations, highlighting the need for multisectoral interventions that address health, environmental, and socioeconomic issues.

To reduce malaria‐related mortality in indigenous areas, interventions should address not only healthcare concerns but also environmental, socioeconomic, and cultural factors [40, 41]. This may include strengthening health infrastructure, improving access to healthcare services, educating communities about malaria prevention and treatment, and monitoring activities such as deforestation and mining [38, 39, 42, 43].

Additionally, the use of tafenoquine has been tested in Brazil with promising preliminary results, showing both efficacy and safety [41, 44]. Tafenoquine is a radical cure medicine for *P. vivax* malaria infections. It is a single dose treatment with the simultaneous ingestion of two tablets. It requires prior testing for G6PD deficiency, because treating G6PD‐deficient patients with tafenoquine can lead to haemolysis and potentially fatal outcomes [45].

More recently it has also been implemented in the Yanomami indigenous territory, which is in the territory of Roraima and Amazonas states in the Amazon Region. The Yanomami territory has suffered since 2022 a major health and humanitarian crisis that has exacerbated malnutrition, limited access to services, and multiple co‐morbidities/co‐infections. To help control malaria infections in this area, the BMoH implemented the treatment with tafenoquine, and it is generating positive results [46, 47].

Given the frequent movement of individuals between endemic and non‐endemic areas within Brazil [48], tafenoquine's use in the Amazon could help mitigate recurring cases that contribute to severe outcomes, particularly in those with compromised health [49], potentially supported by strategies such as Malakit or SMS reminders [50, 51] to ensure the completion of the treatment. However, the logistical challenges associated with the mandatory G6PD deficiency testing cannot be overlooked.

Widespread G6PD testing would be necessary to prevent adverse reactions like haemolytic anaemia [45], making it important to assess whether health systems in this region are prepared to support such testing.

Although tafenoquine implementation may be costly, its single‐dose regimen simplifies administration and adherence in community settings, compared with the traditional primaquine (3 days) plus chloroquine (7 days) [6]. This strategy can reduce not only mortality, but also relapses, severe cases, and hospitalisations [52, 53].

Defining strategies to protect the populations identified in this study can contribute to reducing the number of malaria deaths and ultimately achieving the goal of eliminating malaria deaths by 2030 [4]. Therefore, strengthening surveillance and death investigation is essential, and the incorporation of verbal autopsy strategies by epidemiological surveillance services could provide greater clarity on the causes of those deaths [15].

In addition, considering that active case detection was found to be a major protective factor for malaria deaths and that delayed treatment (> 48 h) was a major risk factor, enhancing and promoting more active searches will contribute to the reduction of the time between the infection and the surveillance system detection, which will reduce deaths. If a malaria case is detected actively, the odds of death are reduced by 53.0% (AOR = 0.47), obviously because active detection provides faster diagnosis. Consequently, the expansion and strengthening of epidemiological surveillance could lower the number of malaria deaths in the Amazon region and help control and reduce malaria cases.

### 4.5 The Extra‐Amazon Region

In the Extra‐Amazon region, preventing deaths due to malaria requires a different approach, as malaria is not endemic there [8]. The risk factors identified for the Extra‐Amazon deaths were associated with high education levels, infections imported from other countries, delayed treatment (> 48 h), and *P. falciparum* and mixed infections.

These associations are mutually consistent and reflect the epidemiological profile of malaria cases in the Extra‐Amazon region. A considerable proportion of affected individuals have higher education levels—a proxy for increased income and travel frequency—which may heighten the risk of exposure in endemic areas and contribute to delayed diagnosis upon return. Many of these cases are associated with travel‐related occupations, and the majority are imported infections. Notably, imported cases from other countries often involve *P. falciparum*, particularly those originating from the African continent [54], where the malaria burden is predominantly attributed to *P. falciparum*.

The fact that most deaths result from infections acquired abroad suggests a demographic of individuals with higher education levels, which can be considered a proxy for better socioeconomic status. Nevertheless, delayed initiation of treatment increases mortality risk by 3.3 times.

Strengthening surveillance systems, particularly in regions such as the southeast where most malaria‐related deaths occur, is critical. Training healthcare professionals in non‐endemic regions to recognise the symptoms of malaria and to differentiate between malaria species is also essential [8, 25, 52].

Improving diagnostic accuracy, especially for cases where malaria is not immediately suspected, could reduce the proportion of deaths coded as unspecified malaria. Expanding diagnostic training programmes in these regions, along with equipping health centres with better testing facilities (with higher availability of rapid diagnostic tests and microscopies), could ensure earlier detection and treatment, reducing mortality.

Urgent action is needed to strengthen malaria case detection in the Extra‐Amazon region of Brazil. Previous warnings have highlighted the risk of severe disease in individuals travelling to areas with active transmission [17, 55].

Moreover, reinforcing travel medicine initiatives, particularly in ports and airports, would be key to addressing the movement of individuals from malaria‐endemic areas [25, 54]. By increasing surveillance and testing travellers who have recently visited endemic regions, it would be possible to identify and treat cases early, preventing the spread and development of severe cases. This approach, combined with improved training and diagnostic resources, would offer a comprehensive strategy to mitigate malaria deaths outside the Amazon.

### 4.6 Recommendations

Updating Sivep‐Malaria and Sinan surveillance systems with variables directed to follow‐up infections is urgent, mainly due to the uncertain timeline for completion of the ‘e‐SUS linha da vida’ project. This update would allow for more comprehensive analyses of malaria infection progression and the identification of complications related to pre‐existing or concurrent health conditions. Incorporating variables such as ‘case progression’ and ‘comorbidities’ into investigation forms would further strengthen surveillance of malaria deaths [20, 56].

Implementing upgrades, however, requires navigating Brazil's tripartite decision‐making process, in which the BMoH must reach consensus with State and Municipal health departments, which could be challenging considering that those modifications would expand investigation forms requiring further and continuous training—making the investigation process longer and more expensive.

Operationally, Sivep‐Malaria is easier to revise because it is managed directly by the National Malaria Program; by contrast, changes to Sinan depend on the Department of Information and Informatics of the SUS (DATASUS), which limits the National Malaria Program's autonomy. Given that Sivep‐Malaria captures more than 99% of reported malaria cases—virtually all within the Amazon—prioritising its upgrade would provide the most immediate benefit.

Strengthening death surveillance and investigation and timely response across Brazil is also urgent. The BMoH must introduce guidelines for investigating malaria‐related deaths to boost malaria deaths' prevention. Without timely and efficient implementation of these guidelines, the current increase in malaria deaths—especially among indigenous populations—may continue to rise [57]. Until an integrated HIS is developed by the BMoH, the implementation of data linkage routines will be essential to monitor and investigate malaria deaths [20].

In addition, the recurrent inconsistencies observed between mortality and epidemiological data systems highlight the urgent need for the BMoH to implement a national strategy for verifying and harmonising data between these sources. Establishing technical routines for cross‐checking causes of death with reported malaria cases could improve the accuracy and reliability of public health information. Even simple descriptive consistency checks can generate valuable insights to support surveillance and policy planning.

Considering what was previously discussed, it is possible to highlight some areas that need urgent investments:

- Adequate training: Healthcare professionals responsible for death registration must receive proper training to accurately recognise and document malaria as a cause of death, both in endemic and non‐endemic areas.
- Improvement in clinical documentation: Clinical documentation in malaria cases should be enhanced, with detailed records of symptoms (e.g., fever, nausea, vomiting, asthenia and fatigue, abdominal pain, jaundice, bleeding, anaemia, kidney failure, among others) [58], comorbidities (e.g., HIV/Aids), co‐infections with common transmissible diseases in Brazil (e.g., vector‐borne, respiratory diseases), and laboratory tests used to ensure accurate attribution of the cause of death. A specialised medical committee should be formed to deeply discuss these matters before including symptoms, comorbidities, and co‐infections fields in the epidemiological forms.
- Education and awareness: Raising awareness among healthcare professionals and the general population about the severity of malaria and the importance of reporting cases and deaths, mostly in the non‐endemic region, is essential to improve the quality of death records.
- Data integration: Integration of health data systems (SIM, Sinan and Sivep‐Malaria) is crucial to improve surveillance of malaria mortality and advance towards the goal of eliminating malaria deaths in Brazil by 2030.

While challenging, eliminating malaria deaths in Brazil by 2030 remains a feasible goal—provided that urgent and sustained investments, multisectoral coordination, and strategic innovations in surveillance and treatment are implemented.

### 4.7 Limitations

Main limitations of this study are related to the effectiveness of the RL process, which is highly dependent on the quality of the data in the databases used [20, 24]. Although the probabilistic RL method can match pairs where names or birth dates are not identical, its performance is directly influenced by the poor accuracy and completeness of the system [24].

Additionally, because the RL process recovered data for only 43.8% of malaria‐related deaths, it is possible that some deaths may remain among controls; however, this represents < 0.02% of the control group and is unlikely to bias results materially. It is also possible that the unmatched deaths were due to the underreporting of malaria cases, meaning that some malaria‐related deaths were not recorded in the Sivep‐Malaria system.

This study was unable to analyse deaths related to malaria recurrences [59] due to the absence of information on this event in the databases. Similarly, data on G6PD deficiencies [60] and comorbidities in the control group [61, 62, 63] were also unavailable. Updates to the malaria HIS may help address those gaps in the future, and additional primary or longitudinal studies could further investigate and quantify how these factors influence malaria‐related mortality in Brazil.

## Conflicts of Interest

The authors declare no conflicts of interest.

## Supporting information


**Data S1:** Data supply letter from the Brazilian Ministry of Health.

**Data S2:** Full logistic regression results.

**Data S3:** Ethics Committee approval.

## Data Availability

Nominal data from this study is considered sensitive and cannot be shared. However, anonymised data can be made available upon reasonable request, subject to the appropriate approvals and data‐sharing agreements.
