# Supplementary material for: Towards Malaria Elimination: A Nationwide Case–Control Study to Assess Risk Factors for Severe Malaria‐Related Deaths in Brazil
Source: Trop Med Int Health. 2025 Sep 24;30(11):1194–210. doi: 10.1111/tmi.70028 (PMC12588806; doi:10.1111/tmi.70028)
Supplement: Supplementary file 1 — Data S1: Data supply letter from the Brazilian Ministry of Health. [file TMI-30-1194-s002.pdf]

Ministério da Saúde
Secretaria de Vigilância em Saúde e Ambiente
Departamento de Análise Epidemiológica e Vigilância de Doenças não Transmissíveis

CONSENT FORM

Reference: Processes Protocol No. 25000.052402/2024-04

This concerns Official Letter No. 11146854/2024 (SEI No. 25000.052402/2024-04) from the University of Brasília requesting access to national databases to conduct the project "Spatio-temporal distribution of malaria deaths with a study of their risk factors and associated causes in Brazil from 2011 to 2020" carried out by researcher Klauss Kleydmann Sabino Garcia and under the coordination of researcher Walter Massa Ramalho.

In this context, access was authorized to anonymized data resulting from the relationship of the following databases: Mortality Information System (SIM), Notifiable Diseases Information System (Sinan), and the Malaria Epidemiological Surveillance System (SIVEP-Malaria) for the period from 2011 to 2020.

The process of relating the databases was carried out within the facilities of the Access Room of this Department of Epidemiological Analysis and Surveillance of Non-Communicable Diseases of the Health Surveillance Secretariat of the Ministry of Health (DAENT/SVSA/MS) so that researchers only had access to the anonymized product of the relationship by signing the Term of Responsibility.

We are available to provide any necessary clarifications.

Geórgia Maria de Albuquerque

Director DAENT/SVSA/MS

Documento assinado eletronicamente por **Geórgia Maria de Albuquerque**, **Diretor(a) do Departamento de Análise Epidemiológica e Vigilância de Doenças não Transmissíveis substituto(a)**, em 09/07/2024, às 14:09, conforme horário oficial de Brasília, com fundamento no § 3º, do art. 4º, do Decreto nº 10.543, de 13 de novembro de 2020; e art. 8º, da Portaria nº 900 de 31 de Março de 2017.

A autenticidade deste documento pode ser conferida no site http://sei.saude.gov.br/sei/controlador_externo.php?acao=documento_conferir&id_orgao_acesso_externo=0, informando o código verificador **0041858283** e o código CRC **2439406F**.

Departamento de Análise Epidemiológica e Vigilância de Doenças não Transmissíveis - DAENT
SRTVN 701, Via W5 Norte Edifício PO700, 6º andar - Bairro Asa Norte, Brasília/DF, CEP 70723-040
Site - saude.gov.br
