# Supplementary material for: Towards Malaria Elimination: A Nationwide Case–Control Study to Assess Risk Factors for Severe Malaria‐Related Deaths in Brazil
Source: Trop Med Int Health. 2025 Sep 24;30(11):1194–210. doi: 10.1111/tmi.70028 (PMC12588806; doi:10.1111/tmi.70028)
Supplement: Supplementary file 2 — Data S2: Full logistic regression results. [file TMI-30-1194-s001.docx]

**SUPPLEMENTARY FILE II**

Supplementary table 1. Associated factors to malaria-related deaths in the Brazilian Amazon region, 2011-2020.

|  |  |  | **UNIVARIATE ANALYSIS MODEL** | | | | **FINAL ADJUSTED MODEL** | | | |
| --- | --- | --- | --- | --- | --- | --- | --- | --- | --- | --- |
|  | **Cases** | **Controls** | **Crude Odds-Ratio** | **CI 95%** | | **p-value** | **Adjusted Odds-Ratio** | **CI 95%** | | **p-value** |
| **Sex:** |  |  |  |  |  |  |  |  |  |  |
| Male **[**ref**]** | 110  (58·5%) | 1,267,940  (61·0%) | - | - | - | - |  |  |  |  |
| Female | 78  (41·5%) | 812,109  (39·0%) | 1·11 | 0·83 | 1·48 | 0·49 |  |  |  |  |
| **Age range:** |  |  |  |  |  |  |  |  |  |  |
| Under 6 months old | 8  (4·3%) | 13,698  (0·7%) | 8·29 | 3·93 | 17·46 | < ·001 | 6·07 | 1·60 | 23·00 | 0·01 |
| 6 to 11 months old | 4  (2·1%) | 17,441  (0·8%) | 3·25 | 1·18 | 9·00 | 0·02 | 0·95 | 0·10 | 8·80 | 0·96 |
| 1 to 4 years old | 15  (8·0%) | 198,811  (9·6%) | 1·07 | 0·60 | 1·90 | 0·82 | 0·94 | 0·29 | 3·10 | 0·92 |
| 5 to 9 years old | 8  (4·3%) | 233,227  (11·2%) | 0·49 | 0·23 | 1·03 | 0·06 | 0·41 | 0·13 | 1·30 | 0·13 |
| 10 to 19 years old | 16  (8·5%) | 477,183  (22·9%) | 0·48 | 0·27 | 0·83 | 0·01 | 0·69 | 0·35 | 1·35 | 0·28 |
| 20 to 39 years old **[**ref**]** | 51  (27·1%) | 723,483  (34·8%) | - | - | - | - | - | - | - | - |
| 40 to 59 years old | 38  (20·2%) | 334,721  (16·1%) | 1·61 | 1·06 | 2·45 | 0·03 | 1·49 | 0·85 | 2·62 | 0·17 |
| 60 years old and over | 48  (25·5%) | 81,355  (3·9%) | 8·37 | 5·64 | 12·41 | < ·001 | 8·56 | 5·01 | 14·65 | < ·001 |
| **Race/Colour of skin:** |  |  |  |  |  |  |  |  |  |  |
| Indigenous **[**ref**]** | 36  (19·1%) | 330,946  (15·9%) | - | - | - | - |  |  |  |  |
| Black | 115  (61·2%) | 1,379,720  (66·3%) | 0·77 | 0·53 | 1·11 | 0·16 |  |  |  |  |
| White | 19  (10·1%) | 153,397  (7·4%) | 1·14 | 0·65 | 1·99 | 0·65 |  |  |  |  |
| **Education level (Years of schooling):** |  |  |  |  |  |  |  |  |  |  |
| None | 30  (16·0%) | 171,357  (8·2%) | 2·76 | 1·80 | 4·24 | < ·001 | 2·05 | 1·21 | 3·46 | 0·01 |
| 1 to 7 years **[**ref**]** | 68  (36·2%) | 1,072,838  (51·6%) | - | - | - | - | - | - | - | - |
| 8 to 11 years | 45  (23·9%) | 458,196  (22·0%) | 1·55 | 1·06 | 2·26 | 0·02 | 1·29 | 0·79 | 2·12 | 0·31 |
| 11 years and over | 7  (3·7%) | 39,789  (1·9%) | 2·78 | 1·27 | 6·04 | 0·01 | 2·25 | 0·80 | 6·32 | 0·12 |
| Not applicable | 25  (13·3%) | 245,531  (11·8%) | 1·61 | 1·02 | 2·54 | 0·04 | 1·92 | 0·62 | 5·97 | 0·26 |
| **Occupation in the last 15 days:** |  |  |  |  |  |  |  |  |  |  |
| Rural/Agriculture **[**ref**]** | 40  (21·3%) | 515,237  (24·8%) | - | - | - | - |  |  |  |  |
| Mining | 17  (9·0%) | 133,312  (6·4%) | 1·64 | 0·93 | 2·90 | 0·09 |  |  |  |  |
| Housing | 24  (12·8%) | 21,542  (1·0%) | 1·44 | 0·87 | 2·38 | 0·16 |  |  |  |  |
| Others | 89  (47·3%) | 1,044,443  (50·2%) | 1·10 | 0·76 | 1·59 | 0·63 |  |  |  |  |
| Travel related | 5  (2·7%) | 43238  (2·1%) | 1·49 | 0·59 | 3·77 | 0·40 |  |  |  |  |
| **Local of infection:** |  |  |  |  |  |  |  |  |  |  |
| Rural area **[**ref**]** | 84  (44·7%) | 1,250,389  (60·1%) | - | - | - | - | - | - | - | - |
| Indigenous Villages | 39  (20·7%) | 323,101  (15·5%) | 1·80 | 1·23 | 2·63 | 0·00 | 2·26 | 1·42 | 3·60 | < ·001 |
| Gold Mining área | 12  (6·4%) | 120,112  (5·8%) | 1·49 | 0·81 | 2·72 | 0·20 | 1·35 | 0·64 | 2·85 | 0·44 |
| Urban área | 29  (15·4%) | 303,633  (14·6%) | 1·42 | 0·93 | 2·17 | 0·10 | 1·46 | 0·89 | 2·39 | 0·13 |
| **Region of infection:** |  |  |  |  |  |  |  |  |  |  |
| Amazon region**[**ref**]** | 178  (94·7%) | 2,016,143  (96·9%) | - | - | - | - | - | - | - | - |
| Imported from another country | 10  (5·3%) | 63,936  (3·1%) | 1·77 | 0·94 | 3·35 | 0·08 |  |  |  |  |
| **Type of detection:** |  |  |  |  |  |  |  |  |  |  |
| Passive detection **[**ref**]** | 164  (87·2%) | 1,554,007  (74·7%) | - | - | - | - | - | - | - | - |
| Active detection | 22  (11·7%) | 493,825  (23·7%) | 0·42 | 0·27 | 0·66 | < ·001 | 0·48 | 0·27 | 0·85 | 0·01 |
| **Treatment opportunity (hours since symptoms onset)** |  |  |  |  |  |  |  |  |  |  |
| <= 48h **[**ref**]** | 44  (23·4%) | 1,154,494  (55·5%) | - | - | - | - | - | - | - | - |
| > 48h | 91  (48·4%) | 769,775  (37·0%) | 3·10 | 2·16 | 4·45 | < ·001 | 2·81 | 1·90 | 4·15 | < ·001 |
| **Diagnosis:** |  |  |  |  |  |  |  |  |  |  |
| *P· vivax* **[**ref**]** | 159  (84·6%) | 1,835,992  (88·3%) | - | - | - | - |  |  |  |  |
| *P· falciparum* or mixed infection | 29  (15·4%) | 243,698  (11·7%) | 1·37 | 0·93 | 2·04 | 0·12 |  |  |  |  |
| Other forms | 0  (0·0%) | 397  (0·0%) | 0·00 | 0·00 | 2·78e+165 | 0·97 |  |  |  |  |

Final model adjustment measures: AIC: 2341; R²: 0,06

Supplementary table 2· Associated factors to malaria-related deaths in the Brazilian extra-amazon region, 2011-2020.

|  |  |  | **UNIVARIATE ANALYSIS MODEL** | | | | **FINAL ADJUSTED MODEL** | | | |
| --- | --- | --- | --- | --- | --- | --- | --- | --- | --- | --- |
|  | **Cases** | **Controls** | **Crude Odds-Ratio** | **CI 95%** | | **p-value** | **Adjusted Odds-Ratio** | **CI 95%** | | **p-value** |
| **Sex:** |  |  |  |  |  |  |  |  |  |  |
| Male **[ref]** | 28  (31·5%) | 1,518  (21·5%) | - | - | - | - |  |  |  |  |
| Female | 61  (68·5%) | 5,533  (78·5%) | 0·60 | 0·38 | 0·94 | 0·03 |  |  |  |  |
| **Age range:** |  |  |  |  |  |  |  |  |  |  |
| Up to 19 years old **[ref]** | 2  (2·2%) | 604  (8·6%) | - | - | - | - |  |  |  |  |
| 20 to 39 years old | 36  (40·4%) | 3,368  (47·8%) | 3·23 | 0·78 | 13·44 | 0·11 |  |  |  |  |
| 40 to 59 years old | 34  (38·2%) | 2,484  (35·2%) | 4·13 | 0·99 | 17·25 | 0·05 |  |  |  |  |
| 60 years old and over | 17  (19·1%) | 596  (8·5%) | 8·61 | 1·98 | 37·45 | 0·001 |  |  |  |  |
| **Education level (Years of schooling):** |  |  |  |  |  |  |  |  |  |  |
| None | 1  (1·1%) | 61  (0·9%) | 2·37 | 0·31 | 18·35 | 0·41 | 4·10 | 0·50 | 33·31 | 0·19 |
| 1 to 7 years | 7  (7·9%) | 1,264  (17·9%) | 0·80 | 0·32 | 1·99 | 0·64 | 1·18 | 0·39 | 3·52 | 0·77 |
| 8 to 11 years **[ref]** | 14  (15·7%) | 2,028  (28·8%) | - | - | - | - | - | - | - | - |
| 11 years and over | 25  (28·1%) | 1,234  (17·5%) | 2·93 | 1·52 | 5·67 | 0·00 | 2·33 | 1·07 | 5·06 | 0·03 |
| Not applicable | 1  (1·1%) | 160  (2·3%) | 0·91 | 0·12 | 6·93 | 0·92 | 1·13 | 0·14 | 8·97 | 0·91 |
| **Region of infection:** |  |  |  |  |  |  |  |  |  |  |
| Autochtonous Brazil **[ref]** | 22  (24·7%) | 4,497  (63·8%) | - | - | - | - | - | - | - | - |
| Imported from another country | 59  (66·3%) | 2,092  (29·7%) | 5·76 | 3·52 | 9·43 | < ·001 | 3·52 | 1·33 | 9·32 | 0·01 |
| **Type of detection:** |  |  |  |  |  |  |  |  |  |  |
| Passive detection **[ref]** | 68  (76·4%) | 4,642  (65·8%) | - | - | - | - |  |  |  |  |
| Active detection | 17  (19·1%) | 1,719  (24·4%) | 0·68 | 0·40 | 1·15 | 0·15 |  |  |  |  |
| **Treatment opportunity: (hours since symtoms onset)** |  |  |  |  |  |  |  |  |  |  |
| <= 48h **[ref]** | 9  (10·1%) | 1,324  (18·8%) | - | - | - | - | - | - | - | - |
| > 48h | 72  (80·9%) | 5,495  (77·9%) | 1·93 | 0·96 | 3·86 | 0·06 | 3·35 | 1·02 | 11·03 | 0·05 |
| **Diagnosis:** |  |  |  |  |  |  |  |  |  |  |
| *P. vivax* **[ref]** | 21  (23·6%) | 4,615  (65·4%) | - | - | - | - | - | - | - | - |
| *P· falciparum* or mixed infection | 64  (71·9%) | 2,337  (33·1%) | 6·02 | 3·67 | 9·88 | < ·001 | 2·67 | 1·01 | 7·10 | 0·05 |
| Other forms | 4  (4·5%) | 100  (1·4%) | 8·79 | 2·96 | 26·08 | < ·001 | 2·55 | 0·29 | 22·46 | 0·40 |

Final model adjustment measures: AIC: 396; R²: 0·114

Supplementary table 3. Associated factors to malaria-related deaths in Brazil, 2011-2020.

|  |  |  | **UNIVARIATE ANALYSIS MODEL** | | | | **FINAL ADJUSTED MODEL** | | | |
| --- | --- | --- | --- | --- | --- | --- | --- | --- | --- | --- |
|  | **Cases** | **Controls** | **Crude Odds-Ratio** | **CI 95%** | | **p-value** | **Adjusted Odds-Ratio** | **CI 95%** | | **p-value** |
| **Age range:** |  |  |  |  |  |  |  |  |  |  |
| Under 6 months old | 8  (2·9%) | 13,740  (0·7%) | 4·86 | 2·36 | 10·04 | < ·001 | 9·13 | 3·51 | 23·72 | < ·001 |
| 6 to 11 months | 4  (1·4%) | 17,456  (0·8%) | 1·91 | 0·70 | 5·22 | 0·20 | 1·49 | 0·20 | 10·96 | 0·70 |
| 1 to 4 years old | 16  (5·8%) | 198,894  (9·5%) | 0·67 | 0·39 | 1·15 | 0·14 | 1·47 | 0·73 | 2·97 | 0·28 |
| 5 to 9 years old | 8  (2·9%) | 233,292  (11·2%) | 0·29 | 0·14 | 0·59 | < ·001 | 0·58 | 0·22 | 1·50 | 0·26 |
| 10 to 19 years old | 17  (6·1%) | 477,582  (22·9%) | 0·30 | 0·18 | 0·50 | < ·001 | 0·64 | 0·33 | 1·25 | 0·19 |
| 20 to 39 years old **[ref]** | 87  (31·4%) | 726,851  (34·8%) | - | - | - | - | - | - | - | - |
| 40 to 59 years old | 72  (26·0%) | 337,205  (16·2%) | 1·78 | 1·31 | 2·44 | < ·001 | 1·59 | 0·92 | 2·76 | 0·10 |
| 60 years old and over | 65  (23·5%) | 81,951  (3·9%) | 6·63 | 4·81 | 9·14 | < ·001 | 9·81 | 5·96 | 16·14 | < ·001 |
| **Race/ Colour of skin:** |  |  |  |  |  |  |  |  |  |  |
| White **[ref]** | 55  (19·9%) | 156,448  (7·5%) | - | - | - | - |  |  |  |  |
| Black | 157  (56·7%) | 1,383,041  (66·3%) | 0·32 | 0·24 | 0·44 | < ·001 |  |  |  |  |
| Indigenous | 36  (13·0%) | 330,981  (15·9%) | 0·31 | 0·20 | 0·47 | < ·001 |  |  |  |  |
| **Education level (Years of schooling):** |  |  |  |  |  |  |  |  |  |  |
| None | 31  (11·2%) | 171,418  (8·2%) | 1·41 | 0·91 | 2·18 | 0·12 |  |  |  |  |
| 1 to 7 years | 75  (27·1%) | 1,074,102  (51·5%) | 0·55 | 0·39 | 0·77 | < ·001 |  |  |  |  |
| 8 to 11 years **[ref]** | 59  (21·3%) | 460,224  (22·1%) | - | - | - | - |  |  |  |  |
| 12 years and over | 32  (11·6%) | 41,023  (2·0%) | 6·09 | 3·96 | 9·36 | < ·001 |  |  |  |  |
| Not applicable | 26  (9·4%) | 245,691  (11·8%) | 0·83 | 0·52 | 1·31 | 0·42 |  |  |  |  |
| **Occupation in the last 15 days:** |  |  |  |  |  |  |  |  |  |  |
| Others **[ref]** | 116  (41·9%) | 1,047,024  (50·2%) | - | - | - | - |  |  |  |  |
| Rural/Agriculture | 49  (17·7%) | 516,025  (24·7%) | 0·86 | 0·61 | 1·20 | 0·37 |  |  |  |  |
| Housing | 27  (9·7%) | 215,648  (10·3%) | 1·13 | 0·74 | 1·72 | 0·57 |  |  |  |  |
| Mining | 22  (7·9%) | 134,025  (6·4%) | 1·48 | 0·94 | 2·34 | 0·09 |  |  |  |  |
| Travel related | 42  (15·2%) | 45,302  (2·2%) | 8·37 | 5·88 | 11·91 | < ·001 |  |  |  |  |
| **Local of infection:** |  |  |  |  |  |  |  |  |  |  |
| Rural área **[ref]** | 84  (30·3%) | 1,250,389  (59·9%) | - | - | - | - | - | - | - | - |
| Indigenous villages | 39  (14·1%) | 323,101  (15·5%) | 1·80 | 1·23 | 2·63 | 0·001 | 2·49 | 1·58 | 3·93 | < ·001 |
| Gold Mining área | 12  (4·3%) | 120,112  (5·8%) | 1·49 | 0·81 | 2·72 | 0·20 | 1·49 | 0·73 | 3·04 | 0·27 |
| Urban area | 29  (10·5%) | 303,633  (14·5%) | 1·42 | 0·93 | 2·17 | 0·10 | 1·57 | 0·97 | 2·55 | 0·07 |
| **Region of infection:** |  |  |  |  |  |  |  |  |  |  |
| Autocthonous Brazil **[ref]** | 200  (72·2%) | 2,020,640  (96·8%) | - | - | - | - |  |  |  |  |
| Imported from another country | 69  (24·9%) | 66,028  (3·2%) | 10·60 | 8·03 | 13·90 | < ·001 |  |  |  |  |
| **Type of detection:** |  |  |  |  |  |  |  |  |  |  |
| Passive search **[ref]** | 232  (83·8%) | 1,558,649  (74·7%) | - | - | - | - | - | - | - | - |
| Active detection | 39  (14·1%) | 495,544  (23·7%) | 0·53 | 0·38 | 0·74 | < ·001 | 0·47 | 0·27 | 0·83 | 0·01 |
| **Treatment opportunity: (hours after symptoms onset)** |  |  |  |  |  |  |  |  |  |  |
| <= 48h **[ref]** | 53  (19·1%) | 1,155,818  (55·4%) | - | - | - | - | - | - | - | - |
| > 48h | 163  (58·8%) | 775,270  (37·1%) | 4·59 | 3·36 | 6·25 | < ·001 | 2·72 | 1·86 | 3·99 | < ·001 |
| **Diagnosis:** |  |  |  |  |  |  |  |  |  |  |
| *P· vivax* **[ref]** | 180  (65·0%) | 1,840,607  (88·2%) | - | - | - | - |  |  |  |  |
| *P· falciparum* or mixed infection | 93  (33·6%) | 246,035  (11·8%) | 3·87 | 3·01 | 4·96 | < ·001 |  |  |  |  |
| Other forms | 4  (1·4%) | 497  (0·0%) | 82·30 | 30·44 | 222·53 | < ·001 |  |  |  |  |

Final model adjustment measures: AIC: 2·404; R²: 0,06;
