# Supplementary material for: Towards Malaria Elimination: A Nationwide Case–Control Study to Assess Risk Factors for Severe Malaria‐Related Deaths in Brazil
Source: Trop Med Int Health. 2025 Sep 24;30(11):1194–210. doi: 10.1111/tmi.70028 (PMC12588806; doi:10.1111/tmi.70028)
Supplement: Supplementary file 3 — Data S3: Ethics Committee approval. [file TMI-30-1194-s003.zip › tmi70028-sup-0003-Supinfo3-2.pdf]

PARECER CONSUBSTANCIADO DO CEP

DADOS DO PROJETO DE PESQUISA

Título da Pesquisa: Distribuição espaço-temporal de óbitos por malária com estudo de seus fatores de risco e causas associadas no Brasil de 2011 a 2020.

Pesquisador: KLAUSS KLEYDMANN SABINO GARCIA

Área Temática:

Versão: 1

CAAE: 51246121.0.0000.5558

Instituição Proponente: Núcleo de Medicina Tropical

Patrocinador Principal: Financiamento Próprio

DADOS DO PARECER

Número do Parecer: 5.008.210

Apresentação do Projeto:

Estudo do tipo caso-controle com componente ecológico transversal geoespacial com utilização de dados secundários. A população de estudo será
de indivíduos registrados no Sistema de Informação de Mortalidade (SIM) classificados como óbitos por malária e indivíduos no Sistema de
Informações Hospitalares (SIH) internados por malária que tenham evoluído a óbito.
Serão analisados óbitos ocorridos em todo o território brasileiro, incluindo região da Amazônia legal que é composta pelos estados do Acre, Amapá,
Amazonas, Mato Grosso, Maranhão, Pará, Rondônia, Roraima e Tocantins e demais estados e Distrito Federal na região extra-amazônica. O
período análise será de 2011 a 2020.
Para manejo, tratamento e análise dos dados serão utilizados os seguintes softwares: R versão 4.0.3, Stata versão 12, Tableau versão 2020.2,
Microsoft Excel 2016, SatScan versão 9.7 e Qgis versão 2.18 e versão 3.16.

Objetivo da Pesquisa:

Identificar fatores prognósticos associados ao risco de morte por malária e seu comportamento espaço-temporal no Brasil de 2011 a 2020.

Avaliação dos Riscos e Benefícios:

Segundo o pesquisador:

Riscos:

Este estudo empregará técnicas e métodos retrospectivos com utilização de informações já coletadas pelo Estado Brasileiro e disponíveis em
sistemas de informação. Dessa forma, a pesquisa oferta riscos mínimos à população de estudo e ao considerar o disposto na Resolução 466/2012 e
na Lei Geral de Proteção de Dados Pessoais - LGPD (LEI Nº 13.709, DE 14 DE AGOSTO DE 2018) esta pesquisa dispõe de riscos relacionados à
garantia do sigilo e a confidencialidade dos dados pessoais dos indivíduos participantes da pesquisa, preservando a privacidade de dados
confidenciais. Para assegurar o sigilo e confidencialidade das informações a serem utilizadas os bancos de dados serão solicitados por meio de
ofícios, enviados à Secretaria de Vigilância em Saúde do Ministério da Saúde, acompanhados de Termos de responsabilidade de utilização dos
dados assinados pelo pesquisador principal da pesquisa. Além disso, as informações sensíveis que permitem identificação do indivíduo serão
utilizadas para união de diferentes bancos de dados e este procedimento será feito de acordo com critérios pré-estabelecidos de segurança da
informação do Ministério da Saúde. Após a etapa de união dos bancos as informações sensíveis não serão mais utilizadas.

Benefícios:

Esta pesquisa não apresenta benefícios diretos individuais a cidadãos brasileiros ou estrangeiros. A realização desta pesquisa apresenta benefícios
indiretos pois fornecerá evidências às organizações públicas do Brasil que subsidiarão o desenvolvimento de políticas públicas em saúde que
gerarão melhorias à saúde da população brasileira, principalmente à saúde da população acometida por malária.

Comentários e Considerações sobre a Pesquisa:

Trata-se de pesquisa de Doutorado do Programa de Pós-Graduação em Medicina Tropical no Núcleo de Medicina Tropical da Universidade de Brasília.

Considerações sobre os Termos de apresentação obrigatória:

Apresentou todos os termos obrigatórios, solicitou dispensa do TCLE.

TCLE: Solicitou dispensa do Termo de consentimento livre e esclarecido, alegando que tal pedido se justifica uma vez que a pesquisa utilizará dados secundários.

Recomendações:

Não existem recomendações.

Conclusões ou Pendências e Lista de Inadequações:

O projeto está adequado em relação às exigências deste Comitê de ética, por isso foi aprovado.

Considerações Finais a critério do CEP:

Após apreciação na reunião dia 29/09/2021 do colegiado CEP/FM o projeto foi aprovado.OBS: De acordo com a Resolução CNS 466/12, nos inciso II.19 e II.20, cabe ao pesquisador elaborar e apresentar ao CEP os relatórios parciais e final do seu projeto de pesquisa. Bem como a notificação de eventos adversos, de emendas ou modificações no protocolo para apreciação do CEP.

Este parecer foi elaborado baseado nos documentos abaixo relacionados:

Tipo Documento	Arquivo	Postagem	Autor	Situação
Informações Básicas do Projeto	PB_INFORMAÇÕES_BÁSICAS_DO_PROJETO_1776937.pdf	19/08/2021 15:02:14		Aceito
Informações Básicas do Projeto	PB_INFORMAÇÕES_BÁSICAS_DO_PROJETO_1776937.pdf	19/08/2021 15:02:12		Aceito
Orçamento	Planilha_de_Orcamento.pdf	19/08/2021 15:01:52	KLAUSS KLEYDMANN SABINO GARCIA	Aceito
Outros	Carta_de_resposta_a_pendencias.pdf	19/08/2021 12:30:40	KLAUSS KLEYDMANN SABINO GARCIA	Aceito
Outros	Curriculos_Lattes_pesquisador_principal_Klauss_Kleydmann_Sabino_Garcia.pdf	19/08/2021 12:28:17	KLAUSS KLEYDMANN SABINO GARCIA	Aceito
Outros	Curriculos_Lattes_Walter_Massa_Ramalho.pdf	19/08/2021 12:27:30	KLAUSS KLEYDMANN SABINO GARCIA	Aceito

Outros	Curriculos_Lattes_Anderson_Coutinho.pdf	19/08/2021 12:27:10	KLAUSS KLEYDMANN SABINO GARCIA	Aceito
Outros	Curriculos_Lattes_AmandaAbrahao.pdf	19/08/2021 12:26:57	KLAUSS KLEYDMANN SABINO GARCIA	Aceito
Outros	Curriculos_Lattes_Ana_Flavia_de_Morais_Oliveira.pdf	19/08/2021 12:25:53	KLAUSS KLEYDMANN SABINO GARCIA	Aceito
Outros	Carta_de_encaminhamento_de_projeto.pdf	19/08/2021 12:24:06	KLAUSS KLEYDMANN SABINO GARCIA	Aceito
Outros	Resumo_estruturado.pdf	19/08/2021 12:19:44	KLAUSS KLEYDMANN SABINO GARCIA	Aceito
TCLE / Termos de Assentimento / Justificativa de Ausência	Dispensa_de_TCLE.pdf	19/08/2021 12:08:38	KLAUSS KLEYDMANN SABINO GARCIA	Aceito
Declaração de Pesquisadores	Declaracao_de_pesquisadores_Walter.pdf	19/08/2021 12:05:35	KLAUSS KLEYDMANN SABINO GARCIA	Aceito
Declaração de Pesquisadores	Declaracao_de_pesquisadores_Klauss.pdf	19/08/2021 12:05:14	KLAUSS KLEYDMANN SABINO GARCIA	Aceito
Declaração de Pesquisadores	Declaracao_de_pesquisadores_Anderson.pdf	19/08/2021 12:05:06	KLAUSS KLEYDMANN SABINO GARCIA	Aceito
Declaração de Pesquisadores	Declaracao_de_pesquisadores_Ana.pdf	19/08/2021 12:04:54	KLAUSS KLEYDMANN SABINO GARCIA	Aceito
Declaração de Pesquisadores	Declaracao_de_pesquisadores_Amanda.pdf	19/08/2021 12:04:42	KLAUSS KLEYDMANN SABINO GARCIA	Aceito
Declaração de concordância	Declaracao_de_concordancia.pdf	19/08/2021 12:04:07	KLAUSS KLEYDMANN SABINO GARCIA	Aceito
Cronograma	Cronograma_de_pesquisa.pdf	19/08/2021 12:02:38	KLAUSS KLEYDMANN SABINO GARCIA	Aceito
Projeto Detalhado / Brochura Investigador	Projeto_detalhado_Brochura_Pesquisador.pdf	19/08/2021 12:02:19	KLAUSS KLEYDMANN SABINO GARCIA	Aceito
Folha de Rosto	Folha_de_rosto_KlaussGarcia.pdf	19/08/2021 11:56:44	KLAUSS KLEYDMANN SABINO GARCIA	Aceito

Situação do Parecer:
Aprovado

Necessita Apreciação da CONEP:
Não

BRASILIA, 30 de Setembro de 2021

Assinado por:
Antônio Carlos Rodrigues da Cunha
(Coordenador(a))

Endereço: Universidade de Brasília, Campus Universitário Darcy Ribeiro - Faculdade de Medicina
Bairro: Asa Norte **CEP:** 70.910-900
UF: DF **Município:** BRASILIA
Telefone: (61)3107-1918 **E-mail:** cepfm@unb.br
